# Pediatric CNS Tumors

**DOI:** 10.1038/sj.bjc.6602135

**Published:** 2004-09-28

**Authors:** M Gaze

**Affiliations:** 1 University College London, UK

This multidisciplinary handbook of childhood brain and spinal cord tumours is well-produced, and illustrated with many high-quality neuro-radiological images demonstrating typical MRI and CT scan appearances, and colour photomicrographs to show tumour histopathology. The layout is modern and clear with two text colours and, many tables and summary boxes make for easy reading.

Following a brief introduction reviewing the aetiology, epidemiology and classification of childhood central nervous system tumours, this book follows a traditional layout. Overall, 10 chapters cover the principal tumour types grouped by anatomical site and pathology. These cover tumour natural history, diagnosis and management principles. There is probably too much coverage of molecular biology observations which are of no current relevance, and too little on the practical detail of treatment.

There follows an excellent chapter describing the most common neurocutaneous syndromes and the CNS tumours with which they can be associated.

The final chapters cover imaging, surgery, chemotherapy and radiotherapy. These may be useful to inform practitioners in other specialties, but do not contain enough detail or practical applications to guide practitioners on the specialty they cover. There is tantalising mention of a number of technological innovations without citation of any evidence base to suggest that they are better than conventional treatment, or any words of caution that they should only be used in the context of a well-designed prospective study.

The book essentially covers the tertiary and quaternary management of childhood CNS tumours in a paediatric neurosurgical and oncology centre. It has 28 contributors, all from the United States, of whom 19 are from San Francisco, California. Despite this restricted authorship, the evidence-based treatment recommendations are very largely those which would be given in any large paediatric neuro-oncology centre in Europe or America. It will be valuable to the specialist trainee or clinical nurse specialist in any relevant discipline.

Sadly however, it contains nothing of relevance to the generalist. For example, nothing in this book will guide a district general hospital paediatrician towards making an early referral for specialist investigation of a previously well child who has developed symptoms or signs which could (but more probably do not) indicate the presence of a brain tumour. Similarly, there is nothing practical to help the general paediatrician in the shared care management of children known to have brain tumours. For example, it would have been useful to have guidance about complications may arise such as hydrocephalus or shunt blockage or infection, and when to refer a child back as an emergency to the neurosurgical centre.

Passing mention is made in different chapters of the complications which may result from chemotherapy or radiotherapy. However, it would have been useful in addition to have a detailed section on supportive care, long-term follow-up and the management of a child who may have complex problems as a result of the tumour and its treatment. These include, but are not limited to, endocrine surveillance and hormone replacement therapy, psychometric testing and educational support, visual and audiological assessment and the support of sight- and hearing-impaired children, neuro-rehabilitation, relief of social and psychological distress in patients and their families, and management of the behaviourally disturbed child.

Rightly, the focus of this book is on curative therapy, but unfortunately cure is not the only outcome. The book would have been enriched by guidance on symptom control and supportive care of the child with progressive, incurable disease, including the appropriate use of corticosteroids, anticonvulsant recommendations, analgesia and antiemetics and palliative interventions with surgery, radiotherapy and chemotherapy.

Despite these clear reservations, this little book will still be useful for many people involved in the care of children with CNS tumours.

